# Tanshinone IIA Suppresses Proliferation and Inflammatory Cytokine Production of Synovial Fibroblasts from Rheumatoid Arthritis Patients Induced by TNF-α and Attenuates the Inflammatory Response in AIA Mice

**DOI:** 10.3389/fphar.2020.00568

**Published:** 2020-05-15

**Authors:** Hongyan Du, Yuechun Wang, Yongchang Zeng, Xiaoming Huang, Dingfei Liu, Lvlan Ye, Yang Li, Xiaochen Chen, Tiancai Liu, Hongwei Li, Jing Wu, Qinghong Yu, Yingsong Wu, Ligang Jie

**Affiliations:** ^1^School of Laboratory Medicine and Biotechnology, Southern Medical University, Guangzhou, China; ^2^Department of Rheumatology and Clinical Immunology, Zhujiang Hospital, Southern Medical University, Guangzhou, China; ^3^School of Chinese Medicine, Southern Medical University, Guangzhou, China

**Keywords:** Tan IIA, suppress, RA-FLSs, AIA, MAPK, AKT/mTOR, HIF-1α

## Abstract

Rheumatoid arthritis (RA) is a chronic and progressive autoimmune disease in which activated RA fibroblast-1ike synoviocytes (RA-FLSs) are one of the main factors responsible for inducing morbidity. Previous reports have shown that RA-FLSs have proliferative features similar to cancer cells, in addition to causing cartilage erosion that eventually causes joint damage. Thus, new therapeutic strategies and drugs that can effectively contain the abnormal hyperplasia of RA-FLSs and restrain RA development are necessary for the treatment of RA. Tanshinone IIA (Tan IIA), one of the main phytochemicals isolated from *Salvia miltiorrhiza* Bunge, is capable of promoting RA-FLS apoptosis and inhibiting arthritis in an AIA mouse model. In addition, RA patients treated at our clinic with Tan IIA showed significant improvements in their clinical symptoms. However, the details of the molecular mechanism by which Tan IIA effects RA are unknown. To clarify this mechanism, we evaluated the antiproliferative and inhibitory effects of proinflammatory factor production caused by Tan IIA to RA-FLSs. We demonstrated that Tan IIA can restrict the proliferation, migration, and invasion of RA-FLSs in a time- and dose-dependent manner. Moreover, Tan IIA effectively suppressed the increase in mRNA expression of some matrix metalloproteinases and proinflammatory factors induced by TNF-α in RA-FLSs, resulting in inflammatory reactivity inhibition and blocking the destruction of the knee joint. Through the integration of network pharmacology analyses with the experimental data obtained, it is revealed that the effects of Tan IIA on RA can be attributed to its influence on different signaling pathways, including MAPK, AKT/mTOR, HIF-1, and NF-kB. Taken together, these data suggest that the compound Tan IIA has great therapeutic potential for RA treatment.

## Introduction

Rheumatoid arthritis (RA) is a chronic and systemic autoimmune disease characterized by deformity and joint dysfunction (Smolen et al., 2016). Although the pathogenesis and etiology of RA have not been fully explained, fibroblast-like synoviocytes (FLSs) are considered to be crucial in the development of synovial hyperplasia and the progressive joint destruction in RA patients (Huber et al., 2006; Lefevre et al., 2009). Recent evidence indicates that activated RA-FLSs display biological characteristics similar to tumor cells, such as aggressive proliferation, migration, and invasion. Remarkably, these features are conducive to causing damage to articular cartilage and bone (Bustamante et al., 2017; Wang Z. et al., 2019; Wang and Zhao, 2019). Therefore, the inactivation of RA-FLSs has been pointed to as a potential therapeutic strategy for the treatment of RA.

Many natural ingredients from herbal medicine have been found to be pharmaceutically effective against RA. *Salvia miltiorrhiza* Bunge, a famous herbal medicine, has been widely used to treat cardiovascular diseases in China. Tanshinone IIA (Tan IIA) is the main phytochemical isolated from *S. miltiorrhiza* and is the main contributor to its beneficial cardiovascular effect. Besides, several studies have revealed other medicinal effects of Tan IIA, including anti-tumor, anti-proliferation, and anti-inflammatory effects in various cancers, such as non-small-cell lung cancer, liver cancer, cervical cancer, colorectal cancer, and gastric cancer (Sui et al., 2017; Zhang et al., 2018; Liu et al., 2019; Wang R. et al., 2019; Zhang et al., 2019). Additionally, there are also reports that Tan IIA can be used to treat arthritis (Jia et al., 2017; Zhang et al., 2017).

RA patients have an increased mortality rate due to cardiovascular events. The increase in inflammation associated with RA is the main mechanism that leads to an increase in the cardiovascular mortality rate. These data may suggest that aggressive treatment of inflammation may decrease cardiovascular risk in patients with RA. Tan IIA has been shown to have anti-inflammatory and immunomodulatory effects on atherosclerosis (Chen and Xu, 2014). Recent studies pointed out that Tan IIA can be used in antiatherosclerosis treatment targeting immune cells, antigens, cytokines, and cell signaling pathways (Ren et al., 2019). In this context, the anti-inflammatory and immunomodulatory effects of Tan IIA could be used in the treatment of rheumatoid arthritis also. In fact, patients with RA treated at our clinic with compound Salvia injection, in which Tan IIA is one of the main ingredients, showed significant improvements in their clinical symptoms (Jie et al., 2002; Jie et al., 2010).

All of the above indicate that Tan IIA is safe and could be a potential clinical medicine, but further research on the mechanism is needed to provide a basis for clinical use. In particular, for RA patients with cardiovascular disease or related risk factors, Tan IIA may be a better choice than the alternatives. In recent years, several studies have focused on the effect and the mechanism of tanshinone in the treatment of RA. Our previous studies demonstrated that Tan IIA induced apoptosis of RA-FLSs by blocking the cell cycle in the G2/M phase and regulating a mitochondrial pathway. In addition, other studies have shown that Tan IIA and a derivate, sodium tanshinone IIA sulfonate, inhibited proliferation, migration, invasion, and inflammation in RA-FLSs and attenuated RA progression in collagen-induced arthritis (CIA) mice (Tang et al., 2019; Wang Z. et al., 2019). However, the details of the molecular mechanisms that result in the effect of Tan IIA on RA have not yet been discovered due to its various effects and targets. Therefore, in this study, several approaches (an AIA animal model for *in vivo* experiments, RA-FLS strain construction for *in vitro* evaluation, and network pharmacology and signaling pathway analyses) were applied to further investigate the effects and therapeutic use of Tan IIA in RA.

## Materials and Methods

### Animals

Male C57BL/6 mice at the age of 10–12 weeks were obtained from the Lab Animal Center of Southern Medicine University. The experiment was approved by the Southern Medical University Ethics Committee for Animal Laboratory Research. All animal experimentation procedures were in accordance with the Ethical Guide for Institutional Animal Care and Use of Laboratory Animals of the National Institutes of Health. The mice were fed in the suitable environment according to previously described conditions (Du et al., 2019).

### AIA Induction and Tan IIA Treatments

**Figure 1:**
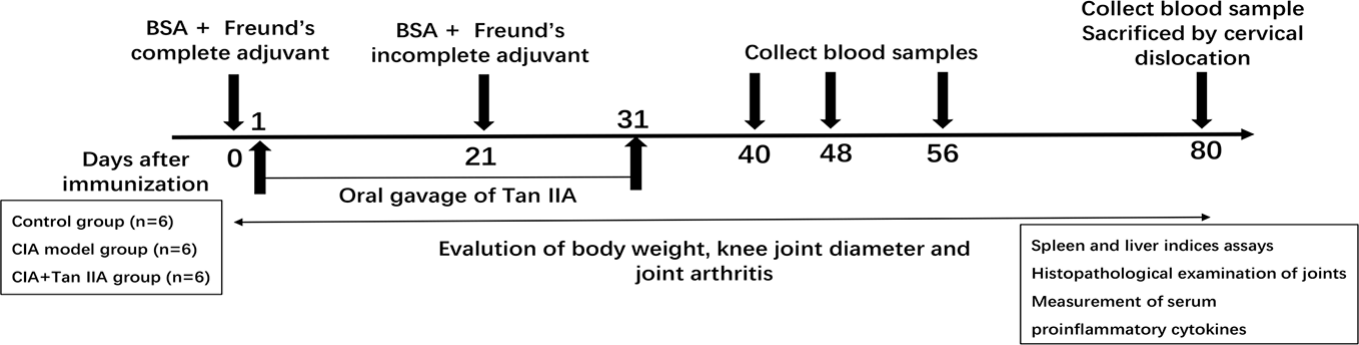
Study design of the AIA experiment. Male C57BL/6 mice aged 10–12 weeks were immunized at each side of the knee articular cavity on day 0, and second immunizations were performed on day 21. From day 2 to day 31 after immunization, mice were administered with oral gavage Tan IIA once a day consecutively, as described in the Materials and Methods. Body weight and knee joint diameter were measured every 5 days. Blood samples for proinflammatory cytokine analysis were collected on days 40, 48, 56, and 80. The mice were euthanized at day 80, and bone, spleen, liver, and serum samples were collected. Histopathological analysis was performed on the bones of knee joints. Spleen and liver indices were calculated by weighing spleen and liver. Serum samples were subjected to ELISA assay.

Eighteen male C57BL/6 mice, about 20 g in body weight each, were divided into three groups randomly: the normal group, AIA model group, and AIA model with Tan IIA treatment group. The protocol for inducing the AIA model was as previously described (Atkinson and Nansen, 2017; Dong et al., 2019; Du et al., 2019; Grötsch et al., 2019), adjusted on some points. The experimental timeline for AIA is shown in **Figure 1**. Briefly, mixtures (1:1/volume ratio) of 5% bovine serum albumin (BSA, Sigma, USA) and Freund's complete adjuvant (CFA) (Sigma-Aldrich, USA) were made by emulsification. On day 0, the mice immunizations were performed by subcutaneously injecting 100 μL of emulgator into the knee joint space under general anesthesia. Mice were injected with 20 μL of emulgator in which Freund's incomplete adjuvant (IFA) (Sigma-Aldrich, USA) was substituted for CFA on day 21. From day 2 to day 31 after immunization, mice were intragastrically administrated with 100 μL Tan IIA (30mg/kg, Selleck, Shanghai, China) every single day. The normal and AIA model groups were given an equal volume of 1% sodium carboxylmethyl cellulose suspension i.g. simultaneously. Body weight and mediolateral knee joint diameter were monitored by experimenters blinded to the experimental design every 5 days (Frey et al., 2018; Dong et al., 2019; Du et al., 2019).

### Measurement of Serum Proinflammatory Cytokine Concentration

On days 40, 48, 56, and 80 after immunization, 200–300 μL blood samples were gathered from the eyeballs of mice and 100–200 μL serum samples were separated by centrifuge and stored at -80℃ for analysis. The ELISA detections of IL-6, IL-17, and TNF-α were carried on with ELISA kits (Jiangsu Meimian Industrial Co., Ltd, Jiangsu, China) according to the manufacturer's instructions (Gou et al., 2018; Du et al., 2019; Li et al., 2019).

### Measurement of Spleen and Liver Indices

On day 80 after immunization, all the mice were sacrificed by cervical dislocation. The liver and spleen indices were determined by the ratio of spleen and liver wet weight to mouse body weight (g/g), respectively. They were expressed as organ index=organ wet weight (g)/animal body weight (g) × 100% (Hu et al., 2005; Gou et al., 2018; Du et al., 2019).

### Histopathological Evaluation of Joints

Hind limbs with knee articular were removed from mice and fixed in Roles-Bio^®^ Universal Tissue Fixative (Roles-Bio, Guangzhou Routh Biotechnology Co., Ltd.). Subsequently, the tissues were decalcified with Roles-Bio^®^ Quick Decalcifying Solution (Roles-Bio, Guangzhou Routh Biotechnology Co., Ltd.) and embedded in paraffin. About 5-μm-thick paraffin sections were made and stained with hematoxylin and eosin (H&E) (Gou et al., 2018; Du et al., 2019). The HE results were graded in a blinded manner according to previous research (Du et al., 2019; Grötsch et al., 2019). The scoring standard was as follows: 1=mild, 2=moderate, and 3=severe.

### Cells Isolation and Culture

The synovial tissues were removed from the knee joints of active RA patients who were undergoing synovectomy with arthroscopy. The detailed data from the patients, of whom 2 were males and 4 females, were shown in **Table S1**. RA patients selected for our research conformed to the American College of Rheumatology revised criteria of the diagnosis of RA (Arnett et al., 1988) and provided informed consent. Moreover, our experiments were in accordance with the guidelines formulated by the Medical Ethics Committee of Zhujiang Hospital, Southern Medical University, and were performed according to the recommendations of the Declaration of Helsinki. The primary synoviocytes (RA-FLSs) were isolated from the harvested synovial tissue and cultured according to our previously published research (Du et al., 2019). After being subcultured, the three to six passage RA-FLSs were used for the subsequent experiments. All reagents for culturing cells were purchased from Gibco^®^ (Thermo Fisher Scientific, MA, USA).

### Cell Viability Assay

RA-FLSs were placed in a 96-well plate and treated with Tan IIA (C_19_H_18_O_3_, ≥98% HPLC, CAS:568-72-9, Selleck) at various concentrations (0 μM, 2.5 μM, 5 μM, 10 μM, 20 μM) and TNF-α (20 ng/mL). The cell viability assay was carried out with a Cell Counting Kit (CCK-8) (KeyGEN BioTECH) according to the manufacturer's instructions. The absorbance was measured at 450 nm with a microplate reader.

### Cell Migration and Invasion Assay

RA-FLS migration and invasion assays were performed in a Boyden chamber with 6.5-mm-diameter inserts containing 8-μm pores (Costar, New York, NY, USA) or coated with Matrigel basement membrane matrix (BD Biosciences, Oxford, UK) in a 24-well plate. Briefly, after being treated with various concentrations of Tan IIA for 24 h respectively, 4×10^3^/200 μL RA-FLSs suspended in serum-free DMEM medium were added into the upper chamber, and 500 μL DMEM media with 10% FBS were placed in the lower well as a chemoattractant. Following incubation, the cells that had migrated through the filter were fixed and stained with 0.1% crystal violet. The cells were quantified by counting the stained cells with a microscope. The mean number of cells per 5–6 random fields was calculated for each assay (Du et al., 2019; Wu et al., 2019).

### Wound Healing Assay

RA-FLSs were planted into a 12-well culture dish on the first day. On the next day, a pipette tip was used to make a scratch, and deciduous cells were washed away with PBS twice. After being treated with various concentrations of Tan IIA for 48 h, the wound areas were photographed with a microscope and the extent of would closure was calculated with Image J software. The data are shown as the mean ± SD of three independent experiments.

### RNA Isolation and Real-Time PCR Assay

Real-time PCR was performed for analyzing the expression of some cytokines and MMPs in RA-FLSs treated with Tan IIA according to previous descriptions (Jie et al., 2015; Du et al., 2019). Total RNAs in RA-FLSs treated with or without TNF-α (20ng/mL) and Tan IIA were isolated by TRIzol (Invitrogen, U.S.A.) and reverse transcribed into cDNA using the Prime Script RT Reagent kit (Takara Biotechnology, Dalian, China), adopting the manufacturer's protocol. According to the manufacturer's instructions, PCR quantification for cytokines and MMP mRNA with an SYBR Premix Ex TaqTM kit (Takara Biotechnology, Dalian, China) was carried out in an ABI 7500 type PCR instrument (Applied Biosystems Inc., Foster City, CA, USA). DdH_2_O containing no template was set as negative control. All of the primers were synthesized by IGE Biotech. Co., Ltd (Guangzhou, China) and are listed in **Table S2**. All experiments were performed in triplicate and repeated three times independently. To quantify the relative expression of each gene, the ΔΔCt method (ΔΔCt =ΔCt_sample_−ΔCt_control_) was used to indicate the ratio of the expression of the target gene in the model group to that of the control group (Du et al., 2019; Wu et al., 2019).

### Western Blot Assay

After treatment with TNF-α (20ng/mL) or/and 10μM and 20 μM Tan IIA for 24 h, RA-FLSs were collected and total protein was extracted using RIPA lysis buffer and phosphatase inhibitors (Beyotime Biotechnology, Nantong, China) on ice. The proteins from RA-FLSs were obtained by separating supernatants and debris *via* centrifugation at 12,000 rpm for 20 min at 4°C. The Pierce^®^ BCA Protein Assay Kit (Thermo Scientific, USA) was used to determine the protein concentration. The levels of protein were adjusted to 0.5–1 μg/μL and detected by automated electrophoresis western analysis assay (ProteinSimple, Biotechne, San Jose CA, United States) as described previously (Baradaran-Heravi et al., 2016). According to the user manual, all procedures were performed using the manufacturer's reagents. Briefly, 8 µl diluted protein lysate was mixed with 2 µl of 5× fluorescent master mix and heated at 95°C for 5 min. Various ingredients, including the sample (about 1 µg), blocking reagent, wash buffer, primary antibodies, secondary antibodies, and chemiluminescent substrate were allotted into the designated wells in a manufacturer-provided microplate. The plate was loaded into the instrument, and protein was drawn into individual capillaries on a 25-capillary cassette provided by the manufacturer (Jess/Wes Separation 12-230 kDa 8×25 Capillary Cartridges kit). Protein separation and immunodetection were automatically performed on the individual capillaries using the default settings. The data were analyzed with inbuilt Compass software (ProteinSimple, Biotechne, United States). The truncated and target protein peak intensities (area under the curve) were normalized to that of the vinculin peak, used as a loading control. Primary antibodies included AKT, mTOR, p70S6K, 4E-BP1, p38 MAPK, p44/42 MAPK (Erk1/2), JNK, NFκB p65, Iκκα, and HIF-1α and their corresponding phosphorylation antibodies, Phospho-Akt (Ser473), Phospho-p70 S6 Kinase (Thr389), Phospho-4E-BP1 (Ser65), Phospho-p38 MAPK(Thr180/Tyr182), Phospho-p44/42 MAPK (Erk1/2) (Thr202/Tyr204), Phospho-JNK (Thr183/Tyr185), p-NFκB p65(Ser 536), and p-Iκκα/β(Ser176/180), which were all purchased from Cell Signaling Technology, USA. GAPDH antibodies used as a reference standard for quantification were purchased from Bioworld Technology Inc.

### Measurements of Cytokine Levels by ELISA

To determine the effect of Tan IIA on cytokine production, ELISA experiments were performed using human enzyme-linked immunosorbent assay (ELISA) kits (Jiangsu Meimian Industrial Co., Ltd, Jiangsu, China) according to the manufacturer's instructions. For example, RA-FLSs were seeded into six-well plates and treated with TNF-α (20ng/mL) or/and 10μM and 20μM Tan IIA for 48 h. The culture supernatants were collected, and the level of IL-6 release from the RA-FLSs was detected as previously described (Jie et al., 2015; Du et al., 2019). The other cytokine assays were carried out using the same method. All experiments were performed in triplicate and replicated 3 times.

### Search for Potential Tan IIA Targets in RA by Network Pharmacology

Firstly, data preparation was carried out by searching for Rheumatoid Arthritis-related genes at the National Biotechnology Center (https://www.ncbi.nlm.nih.gov). Additionally, the chemical structure, molecular weight, 2D structure, 3D structure, chemical number, and physicochemical properties of Tan IIA had to be confirmed. The target genes of Tan IIA were obtained by PharmMapper (http://www.lilab-ecust.cn/pharmmapper/). Next, a drug–target–disease interaction network was constructed. A Venn diagram was constructed based on the functions of the human genes related to rheumatoid arthritis and the potential Tan IIA targets, and the intersection target genes were obtained. Moreover, a protein–protein interaction network (PPI) was constructed on-line by STRING (https://string-db.org/cgi/input.pl). Finally, biological process and pathway analysis was performed. According to the function of human genes related to rheumatoid arthritis and potential Tan IIA targets, the Bioconductor database was used to perform Gene Ontology (GO) Enrichment and Kyoto Encyclopedia of Genes and Genomes (KEGG) pathway enrichment analysis of target genes through R (R 3.6.1 for Windows). The target genes were screened with P < 0.05 set as the critical value of significant functions and pathways, and the main signaling pathways and biological processes involved in the pharmacological effects of Tan IIA in treating rheumatoid arthritis were obtained.

### Statistical Analysis

Data from multiple experiments are presented as the mean ± standard deviation (SD). Statistical software was used for all data analysis. The statistical difference comparisons (P-values) between two groups were calculated using Student's t-test, and P-values between more than three groups were calculated using one-way analysis of variance (ANOVA) with GraphPad Prism 8.0. Two-sided p < 0.05 was considered statistically significant. The number of replicates and/or total number of animals are shown in figure legends or within the figures.

## Results

### Tan IIA Attenuates the Inflammatory Response in Mice With AIA

#### Tan IIA Suppresses Weight Loss and Knee Joint Swelling in AIA Mice

**Figure 2:**
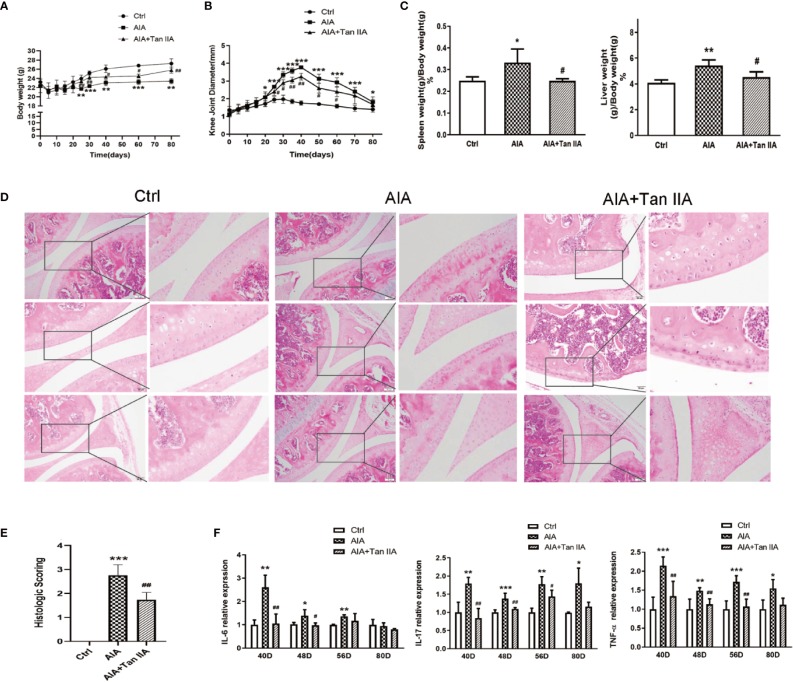
Tan IIA ameliorates arthritis severity in mice with AIA. **(A)** The effect of Tan IIA on mean change in the body weight of mice after immunization (from day 0 to day 80), monitored every 5 days. **(B)** The effect of Tan IIA on mean change in knee joint diameter after immunization (from day 0 to day 80), monitored every 5 days. **(C)** The effect of Tan IIA on the spleen and liver indices of mice with AIA and control. Data are shown as spleen weight (g)/body weight (g) ×100%. **(D)** The effect of Tan IIA on the pathohistological features of knee joints in mice with AIA. Photomicrographs of knee joint sections stained with H&E (original magnification 200×). **(E)** The scores for inflammatory severity. **(F)**The effect of Tan IIA on IL-6, IL-17, and TNF-α expression in serum of mice with AIA and control on days 40, 48, 56, and 80 after immunization. All of the data are expressed as means ± S.D. n=6, ^*^P < 0.05, ^**^P < 0.01, ^***^P < 0.001 vs. the control group, ^#^*P* < 0.05, ^##^*P* < 0.01, vs. the AIA model group.

All of the mice from different groups could access food and water freely during the whole study period. To clarify the effect of Tan IIA on AIA model mice, the mean changes in body weight of mice were monitored every 5 days from day 0 to day 80. As shown in **Figure 2A**, the mean body weight change of mice from the AIA group significantly decreased compared with the change in the normal group at the 25^th^ day after immunization. Nevertheless, compared with the normal group, the mean body weight change of mice from the group treated with Tan IIA (30mg/kg) *via* gavage had declined little at that time. There was significant difference between the Tan IIA treatment group and the AIA model group.

Synchronously, the effect of Tan IIA on arthritis severity, as characterized by measurements of the knee joint diameters, were assessed every 5 days. As shown in **Figure 2B**, the mean value of the diameters of knee joints in AIA mice increased obviously compared to the normal group from the 20^th^ day after immunization because of obvious swelling. Moreover, the increase was rapid from the 25^th^ to the 40^th^ day, when the mean diameter reached a peak value. After the 40^th^ day, it gradually reduced. The values of the mean diameter for AIA mice were significantly different from those of the normal control group during the whole process. However, the mean diameter of knee joints in the mice with Tan IIA treatment was lower than that of AIA mice from the 25^th^ to the 60^th^ day.

#### Tan IIA Reduces Spleen and Liver Indices of AIA Mice

The spleen and liver indices from mice in different groups were assessed to evaluate the effect of Tan IIA on the main immune organs. The spleen and liver indices of mice in the AIA model group was obviously raised compared with those of the normal group (**Figure 2C**). Nevertheless, the spleen and liver indices for the Tan IIA treatment group were significantly lower than those of the AIA group.

#### Tan IIA Improves the Pathohistological Characters of Knee Joints and Arthritis Severity in AIA Mice

To study how Tan IIA affected the pathohistological features of AIA mice, histological examinations of tissue sections were performed. The knee joints from all mice were removed at the 80^th^ day after euthanization and were then stained with H&E to make pathohistological sections. Clear and complete histological architecture was seen through microscopic observation of the knee joints from the normal control group. However, the knee joints of the AIA model group had abnormal histological architecture, characterized by synovial tissue hyperplasia and massive inflammatory cell infiltration, accompanied by epithelial cell degradation and angiogenesis (microvessel density increase). Compared with the AIA group, the abnormalities of the histological architectures of knee joints from the Tan IIA treatment group were milder, with less synovial hyperplasia, inflammatory cell infiltration, and synovial tissue erosion (**Figure 2D**). Additionally, as shown in **Figure 2E**, the pathohistological score exhibited similar differential tendencies in the three experimental groups, which suggested that Tan IIA did attenuate the inflammatory response in mice from the AIA group and had good anti-arthritic effect.

#### Tan IIA Restrains Proinflammatory Cytokine Expression in AIA Mice

On days 40, 48, 56, and 80 after immunization, the expressions of IL-6, IL-17, and TNF-α in serum from AIA mice with and without Tan IIA treatment were examined by ELISA to explore how Tan IIA affected the proinflammatory cytokines. In the case of IL-6, its expression in serum from AIA mice was significantly higher than in serum from the normal control group on the 40^th^, 48^th^, and 56^th^ day. Moreover, it was obviously increased compared to in mice treated with Tan IIA on days 40 and 48. However, on days 56 and 80, there was no obvious difference between them. Similar trends on days 40, 48, and 56 were observed in IL-17 and TNF-α expression in the three groups. Although there was a difference between the expression levels in normal mice and AIA mice on day 80, no differences in IL-17 and TNF-α were witnessed between the Tan IIA treatment group and the AIA group (**Figure 2F**). All the data indicated that Tan IIA (30mg/kg) could suppress production of the proinflammatory cytokines IL-6, IL-17, and TNF-α in serum of AIA mice.

### Tan IIA Suppresses the Migration and Invasion of RA-FLSs

**Figure 3:**
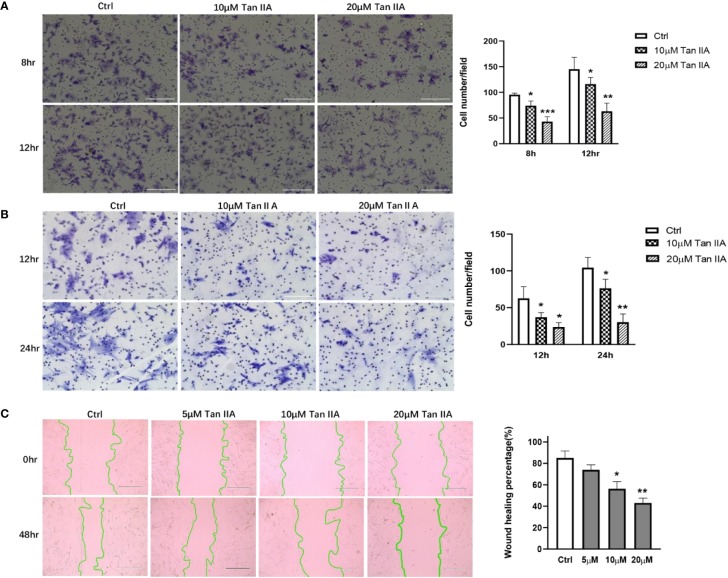
Tan IIA suppresses the migration and invasion of RA-FLSs. **(A)** The effect of Tan IIA on migration was detected with transwell Boyden chamber after 8 and 12 h. The images are representative of migration or invasion through the membrane after staining. Original magnification 200× (left panel). Cell numbers/field are presented as the mean ± SD of eight independent fields (right panel). **(B)** The effect of Tan IIA on invasion was detected with a transwell Boyden chamber coated with a Matrigel basement membrane matrix after 12 and 24 h. The images are representative of migration or invasion through the membrane after staining. Original magnification 200× (left panel). Cell numbers/field are presented as the mean ± SD of eight independent fields (right panel). **(C)** The effect of Tan IIA on wound healing was detected with cell scratch assay. After 48 h, the wound area was photographed using a microscope. Original magnification 100× (left panel). The extent of wound closure is presented as the percentage by which the original scratch width had decreased at each measured time point. The values are the mean ± SEM from at least 3 independent experiments (right panel). ^*^P < 0.05, ^**^P < 0.01, ^***^P < 0.001 vs. 0 μM (Ctrl).

Primary RA-FLSs were separated from synovial tissue from clinical samples. Transwell experiments were performed using the transwell Boyden chamber with or without Matrigel matrix to evaluate the effect of Tan IIA on the migration and invasion of RA-FLSs *in vitro*. Treatment with 10 μM or 20μM Tan IIA profoundly reduced both the migratory and the invasion ability of RA-FLSs comparing with control, as presented in **Figures 3A, B**. This result was further confirmed by wound closure assay, the results of which are shown in **Figure 3C**. After 48 h, the control group cells had almost recovered from the scratch. The cells treated with Tan IIA had inhibited wound healing. Although 5 μM Tan IIA did not significantly interfere with the capacity of RA-FLSs to migrate from one side of the wound to the other, higher concentrations Tan IIA (10 and 20 μM) did restrain the cell migration into the wounded area, as presented in **Figure 3C**. All of the data indicated the Tan IIA could block the migration and invasion of RA-FLSs *in vitro*.

### Tan IIA Inhibits the Viability of RA-FLSs Activated by TNF-α

**Figure 4:**
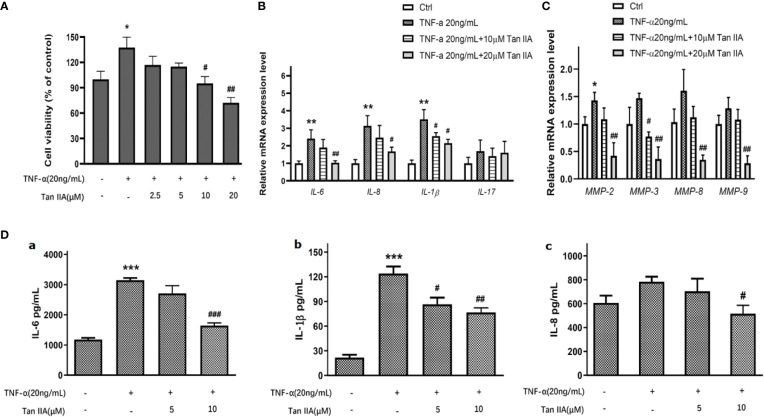
The effect of Tan IIA on producing pro-inflammatory cytokines and MMPs in RA-FLSs. **(A)** The effect of 0, 2.5, 5, 10, and 20μM Tan IIA on cell viability induced by TNF-α (20 ng/mL). **(B)** The effect of 10 μM and 20 μM Tan IIA on relative mRNA expression of pro-inflammatory cytokines induced by TNF-α (20 ng/mL) normalized with β-actin in RA-FLSs. **(C)** The effect of 10 μM and 20 μM Tan IIA on relative mRNA expression of MMP-2, MMP-3, MMP-8, and MMP-9 induced by TNF-α (20ng/mL) compared to β-actin in RA-FLSs. **(D)** The effect of Tan IIA on pro-inflammatory cytokines release induced by TNF-α in RA-FLSs. a. The effect of 10 μM and 20 μM Tan IIA on IL-6 release induced by TNF-α (20ng/mL). b. The effect of 10 μM and 20 μM Tan IIA on IL-1β release induced by TNF-α (20ng/mL). c. The effect of 10 μM and 20 μM Tan IIA on IL-8 release induced by TNF-α (20ng/mL). The values are the mean ± SEM from at least 3 independent experiments. ^*^P < 0.05, ^**^P < 0.01, ^***^P < 0.001 vs. Ctrl (0 μM Tan IIA and 0 ng/mL TNF-α). ^#^*P* < 0.05, ^##^*P* < 0.01, ^###^*P* < 0.001 vs. group treated with TNF-α (20 ng/mL).

As is well known, TNF-α is one of the important pro-inflammatory cytokines conducive to RA-FLS survival and progressive arthritis in RA pathology (Bottini and Firestein, 2013; Bustamante et al., 2017). To discover the effect of Tan IIA on the viability of RA-FLSs induced by TNF-α, the effect of Tan IIA with a series of concentrations (0, 2.5, 5, 10, and 20 μM) on the viability of RA-FLSs activated with TNF-α was measured. A concentration of 20 ng/mL TNF-α obviously promoted the viability of RA-FLSs (**Figure 4A**). Tan IIA had almost no effect on cell viability induced by TNF-α after 24 h treatment (data not shown), while higher concentrations Tan IIA (10 and 20 μM) showed a dose-dependent inhibition in cell viability induced by TNF-α after 48 h treatment (**Figure 4A**).

### Tan IIA Suppresses the Pro-inflammatory Cytokine and MMP Expression Stimulated by TNF-α

Accumulating evidence has pointed out that, during the development of RA, certain pro-inflammatory cytokines and matrix metalloproteinases (MMPs) in particular contribute to the pathogenic factors for proliferation, migration, and invasion of RA-FLSs and even erosion of cartilago articularis (Bottini and Firestein, 2013; Bustamante et al., 2017). To explore the role of Tan IIA on the expression of key pro-inflammatory cytokines induced by TNF-α, the mRNA expression levels of *IL-6, IL-8, IL-17*, and *IL-1β* stimulated by TNF-α in RA-FLSs treated with 10 μM and 20 μM Tan IIA for 24 h were assessed with qPCR. As presented in **Figure 4B**, although TNF-α (20ng/mL) did upregulate, to a greater or lesser extent, the mRNA levels of *IL-6, IL-1β*, and *IL-8* in RA-FLSs, 20 μM Tan IIA inhibited the *IL-6, IL-1β*, and *IL- 8* mRNA upregulation stimulated by 20-ng/mL TNF-α, while 10 μM Tan IIA had no obvious effect except for on *IL-1β*. Unexpectedly, neither Tan IIA treatment nor TNF-α stimulation profoundly changed *IL-17* mRNA expression. Additionally, as shown in **Figure 4C**, only an increase in *MMP-2* mRNA expression was induced by TNF-α (20ng/mL), and *MMP-3* mRNA expression was decreased by 10 μM Tan IIA treatment. However, the mRNA expressions of *MMP-2, MMP-3, MMP-8*, and *MMP-9* dropped significantly after 20-μM Tan IIA treatment, which suggested that Tan IIA significantly blocked upregulation in mRNA expression of *MMP-2, MMP-3, MMP-8*, and *MMP-9* stimulated by TNF-α in RA-FLSs.

In addition, the effect of Tan IIA on the release of some pro-inflammatory cytokines stimulated by TNF-α, as well as on the mRNA level, was also investigated. After being treated with Tan IIA (10 μM and 20 μM) for 48 h, ELISA assays for IL-6, IL-1β, and IL- 8 were performed in cell culture supernatant. As indicated in **Figure 4D**, 20-ng/mL TNF-α significantly increased IL-6 and IL-1β production in RA-FLSs, but Tan IIA treatment could suppress the increase, as shown in **Figures 4D-a** and **4D-b**. Of interest, stimulation with 20-ng/mL TNF-α did not arouse profound upregulation of IL-8, but 20 μM Tan IIA indeed downregulated IL-8 release (**Figure 4D-c**). There was no detectable IL-17 in the ELISA assay because there was less expression in cell culture supernatants. In short, the results suggest that Tan IIA may be helpful for reducing the production and release of some MMPs and pro-inflammatory cytokines from RA-FLSs.

### Potential Targets for Tan IIA in RA Found by Database Search Tools

**Figure 5:**
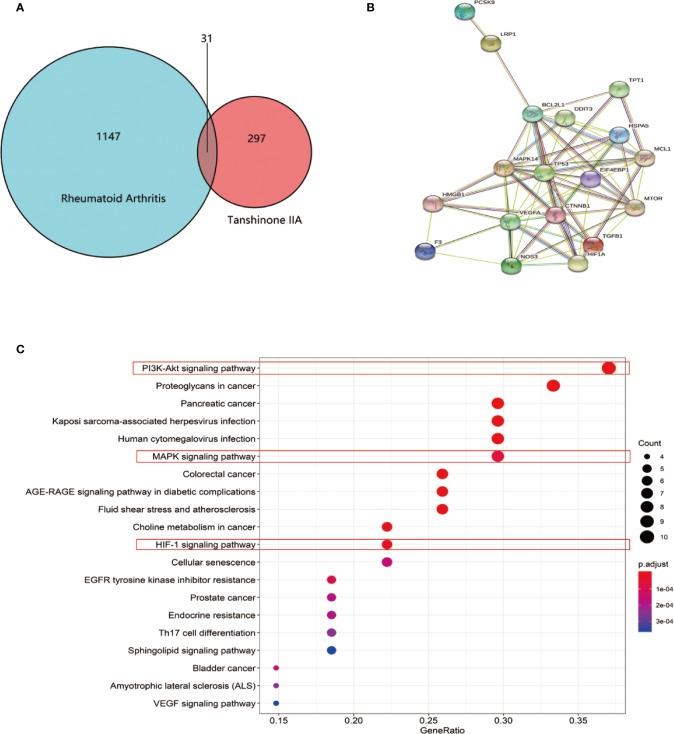
Enrichment analysis of Tan IIA against rheumatoid arthritis: **(A)** Venn diagram revealing the overlapping target genes of Tan IIA against rheumatoid arthritis. **(B)** Protein interaction network of the overlapping target genes of Tan IIA against rheumatoid arthritis. Each network node represents all the proteins produced by a single, protein-coding gene locus, and the edge represents protein–protein associations. **(C)** KEGG enrichment and network analysis of RA target genes. Top 20 functionally enriched biological processes with corresponding adjusted *p*-values, displayed in a dot plot. The color scale indicates the different thresholds of adjusted p-values, and the sizes of the dots represented the gene count of each term.

To uncover potential targets for Tan IIA in RA, we searched the NCBI database and obtained 1147 human genes associated with rheumatoid arthritis. At the same time, we found 297 target genes involved in Tan IIA from the PubChem and PharmMapper databases. A Venn diagram was made with R (R 3.6.1 for Windows) based on the 297 drug targets of Tan IIA and 1147 gene targets of rheumatoid arthritis (**Figure 5A**). We found 31 common targets, which were designated as the key targets of Tanshinone IIA in the treatment of RA. The common targets were then imported into STRING to build the PPI network (**Figure 5B**). This network consists of 71 nodes. The size of the node in the figure indicates the magnitude of the Degree value. The higher the Degree value, the larger the node. We predicted that the proteins BCL2L1, MAPK14, CTNNB1, TP53, EIF4EBP1, HIF1a, HMGB, and mTOR would be potential direct targets of Tan IIA in the treatment of rheumatoid arthritis.

Meanwhile, considering the common targets of RA and Tan IIA, 43 biological processes (P < 0.05) were screened by GO, including protein heterodimerization activity, growth factor activity, receptor regulator activity, disordered domain-specific binding, ribonucleoprotein complex binding, receptor ligand activity, etc. Next, we performed functional enrichment analysis using the KEGG database to clarify the functions of these target genes and signaling pathways. It is of note that the data show that the potential target genes found were functionally related with various signal transduction pathways, including the PI3K-Akt signaling pathway, proteoglycans in cancer, pancreatic cancer; Kaposi sarcoma-associated herpesvirus infection, human cytomegalovirus infection, the MAPK signaling pathway, choline metabolism in cancer, and the hypoxia-inducible factor (HIF-1) signaling pathway (**Figure 5C**). In general, Tan IIA may participate in these pathways, and this could ultimately affect the progression of the disease.

### Tan IIA Affects the Activation of RA-FLSs Induced by TNF-α Through Modulation of the MAPK, Akt/mTOR, and HIF-1 Pathways

**Figure 6:**
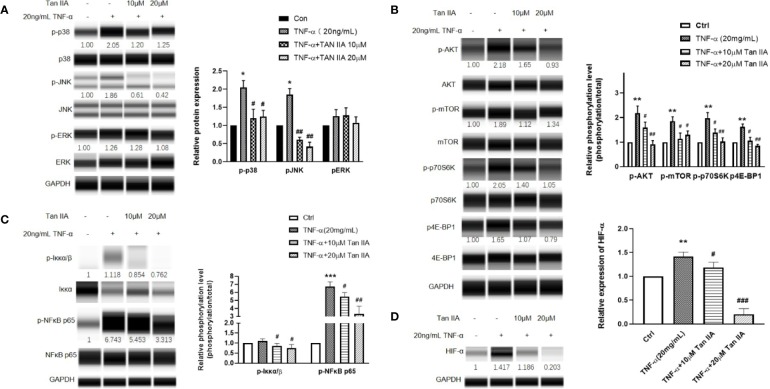
The effect of Tan IIA on the intracellular phosphorylated activation of the MAPK and Akt/mTOR pathway induced by TNF-α in RA-FLSs. RA-FLSs were treated with TNF-α (20ng/mL) or/and Tan IIA (10 and 20 μM) for 24 h. **(A)** Western blot analysis was conducted to assess the expression and phosphorylation levels of p38MAPK, JNK, ERK, and FAK. Representative images of immune blot (left panel) and densitometric quantification phosphorylation/total of p38MAPK, JNK, and ERK expression (right panel). **(B)** Western blot analysis was conducted to assess the expression and phosphorylation levels of AKT, mTOR, p70S6K, and 4E-BP1. Representative images of immune blot (left panel) and densitometric quantification phosphorylation/total of AKT, mTOR, p70S6K, and 4E-BP1 expression (right panel). **(C)** Western blot analysis was conducted to assess the expression and phosphorylation levels of Iκκα and NFκB p65. Representative images of immune blot (left panel) and densitometric quantification phosphorylation/total of Iκκα and NFκB p65 expression (right panel). **(D)** Western blot analysis was conducted to assess the expression level of HIF-α. Representative images of immune blot (left panel) and densitometric quantification of HIF-α expression (right panel). Densitometry analysis from three independent experiments was used to quantitate the protein expression. ^*^P < 0.05, ^**^P < 0.01, ^***^P < 0.001 vs. Ctrl (0 μM Tan IIA), ^#^*P* < 0.05, ^##^*P* < 0.01, ^###^*P* < 0.001 vs. group treated by TNF-α (20 ng/mL).

Combining the results from GO and KEGG with our preliminary research data, we speculated that Tan IIA probably affected RA through the PI3K-Akt, MAPK, and HIF-1 signaling pathways. We detected the main protein expressions and phosphorylation levels of the MAPK signaling pathway, including of p38MAPK, JNK, and ERK, to further verify our supposition as to the effect of Tan IIA on MAPK. After treatment with 20 ng/mL TNF-α and Tan IIA (10 and 20 μM) for 24 h, the RA-FLSs were collected, and the expression and phosphorylation levels of p38MAPK, JNK, and ERK were evaluated by Western blot analysis. As presented in **Figure 6A**, enhanced p38MAPK and JNK phosphorylation activations in RA-FLSs were observed to be induced by TNF-α compared with the control without TNF-α stimulation. Also, Tan IIA efficiently inhibited TNF-α-induced phosphorylation of p38MAPK and JNK. Intriguingly, Tan IIA had less influence on the ERK phosphorylation level. The fact that Tan IIA strongly reduced p38MAPK and JNK activity may contribute to controlling abnormal synovial hyperplasia in the articular cavity.

Moreover, the phosphorylation levels of the Akt/mTOR signaling pathway and its downstream molecules, p70 ribosomal S6 kinase (p70S6K) and eukaryotic translation initiation factor 4E-binding protein 1 (4E-BP1), in RA-FLSs were evaluated with Western blot to explore the effect of Tan IIA on the mTOR pathway. From **Figure 6B**, it can be seen that Tan IIA indeed inhibited the phosphorylation activation of Akt and mTOR stimulated by 20-ng/mL TNF-α. Meanwhile, the increased phosphorylation of p70S6K and 4E-BP1 triggered by TNF-α was also inhibited by Tan IIA treatment in a concentration-dependent manner, suggesting that Tan IIA suppressed the Akt/mTOR/p70S6K and 4E-BP1 signaling pathway in RA-FLSs.

Additionally, according to the results of GO and KEGG analysis, the HIF-1 pathway is a potential target for Tan IIA. The molecular mechanism of hypoxia sensitivity involves oxygen sensing hydroxylases, prolyl-hydroxylases, orchestrating two main transcription factors related to the induction of inﬂammation and angiogenesis, namely nuclear factor-κB (NFκB) and HIF-1 (D'Ignazio and Rocha, 2016; Fearon et al., 2016). Therefore, we detected the effect of Tan IIA on the HIF-1α and NFκB expression variation in RA-FLSs. Similarly, Tan IIA suppressed the phosphorylation level of NFκB p65 and upstream Iκκα (**Figure 5C**) and the HIF-1α expression (**Figure 5D**) stimulated by TNF-α, which indicated that Tan IIA could participate in regulating the response of synovial tissues to hypoxia. Altogether, the regulation of the biological characteristics of RA-FLSs by Tan IIA is dependent on impeding not only intracellular phosphorylation activation of the MAPK and Akt/mTOR pathways but the expression and activation of HIF-1α and NFκB.

## Discussion

RA is a chronic autoimmune disease with a hyperplastic, aggressive, and invasive phenotype that causes the formation of pannus angiogenesis, inflammation, cartilage degradation, and subsequent bone erosion (Smolen et al., 2016). RA-FLSs play a leading role in the pathogenesis of inflammatory arthritis due to their tumor-like features of proliferation, migration, and invasion (Karami et al., 2019). In this context, RA-FLSs stand out as a potential target for RA treatment (de Oliveira et al., 2019). Currently, the main RA treatment strategies in clinical practice are chemical drugs, including non-steroidal anti-inflammatory drugs (NSAIDs), disease-modifying anti-rheumatic drugs (DMARDs), and glucocorticoids (Conigliaro et al., 2019). Nevertheless, these treatments are usually associated with adverse reactions, such as cardiovascular and gastrointestinal bleeding risk, liver and kidney toxicity, growth inhibition, infection, and risk of tumors (Yamamoto et al., 2011; Rubbert-Roth and Petereit, 2012; Xue et al., 2016; Nissen, 2017; Wang et al., 2018). In recent years, progress in research on the pathogenesis of RA has resulted in the development of new anti-rheumatic drugs, such as biological agents and small-molecule targeted signaling pathway inhibitors. These new drugs have greatly improved the chronic inflammatory state and quality of life of RA patients (Conigliaro et al., 2019). However, clinical data show that only less than 50% of RA patients can benefit from these new drugs. Unfortunately, more than 30% of patients still suffer from unsatisfactory disease control, and it is not possible to effectively control disease activities in more than 20% of RA patients. In such cases, the bone destruction process cannot be blocked or delayed, even after the clinical use of these recent drugs (Ranganath et al., 2015; Smolen et al., 2016; Conigliaro et al., 2019).

Recently, herbal medicines have received a large amount of scientific attention for their remarkable healing effects and for having fewer side effects than synthetic drugs. The therapeutic effects of Tan IIA, a compound isolated from *Salvia miltiorrhiza* Bunge *(Salviae miltiorrhizae)*, includes pro-apoptotic, anti-tumor, and anti-inflammatory activities. Additionally, Tang et al. showed that Tan IIA injections could inhibit the inflammatory response in PBMCs of RA patients by decreasing TNF-α and IL-6 levels (Tang et al., 2019). Therefore, the application of Tan IIA in the treatment of RA is feasible in terms of therapeutic effect. To highlight the potential of Tan IIA for RA treatment, we first used an AIA mouse model to verify its therapeutic effects. The AIA model has been widely used to clarify the pathogenesis of RA and to explore potential therapeutic targets, including the validation of the therapeutic effects of new drugs (Sardar and Andersson, 2016; Atkinson and Nansen, 2017; Dong et al., 2019; Du et al., 2019). Our experiments showed that AIA mice treated with Tan IIA showed decreased histologic scores and attenuated synovial inﬂammation. The level of the inflammatory cytokines, including IL-6, IL-17, and TNF-α, measured after 40 days of treatment was significantly higher in the AIA model group than in the normal group. However, the level of inflammatory cytokines was significantly lower in AIA mice treated with Tan IIA than in the AIA model group. The data obtained using the AIA model showed that Tan IIA not only reduced the swelling of the knee joint caused by inflammation but also inhibited the expression of pro-inflammatory factors and improved pathological manifestations in AIA mice. These data corroborate our initial hypothesis that Tan IIA has therapeutic potential for RA treatment. To date, few *in vivo* studies on the effects of Tan IIA in RA treatment have been conducted, and no detailed related mechanisms had previously been discovered.

To discover the mechanisms involved in the effects of Tan IIA on RA, we constructed primary RA-FLS strains from samples of synovial tissue from RA patients. We demonstrated that Tan IIA can inhibit the tumor-like proliferation characteristics of RA-FLSs in clinically safe concentrations. According to our data, although Tan IIA does not have a remarkable effect on the vitality of RA-FLSs after 24-h treatment, it can prevent TNF-α-stimulated cell proliferation in a dose-dependent manner after 48 h of treatment. In addition, previous reports suggested that high concentrations Tan IIA can promote RA-FLS apoptosis (Jie et al., 2014; Li et al., 2018), probably by upregulating lncRNA GAS5 (Li et al., 2018). However, we found in our experiments that RA-FLSs do not undergo apoptosis when treated with up to 20 μM of Tan IIA, while cell apoptosis may accrue at concentrations of Tan IIA above 40 μM. Therefore, we speculate that the effect of Tan IIA on RA-FLSs is different for higher and lower concentrations of Tan IIA, although further studies are needed to elucidate this issue. Moreover, Tan IIA could restrict the migration and invasion of RA-FLSs, which would be better for suppressing the tumor-like properties of RA-FLSs and reducing the damage to distal cartilage.

The RA pathogenesis states that RA-FLSs usually secrete pro-inflammatory factors and chemokines, including TNF-α, IL-6, IL-8, IL-17, and IL-1β, to recruit and activate various immune cells. These immune cells, in turn, secrete cytokines to activate RA-FLSs, contributing to cartilage damage and joint destruction (Bartok and Firestein, 2010; Bottini and Firestein, 2013). TNF-α is one of the most important inflammatory cytokines in the joint cavity of RA patients and is commonly used as an activator of RA-FLSs *in vitro* to simulate the inflammatory microenvironment (Shi et al., 2018; Du et al., 2019; Wang Z. et al., 2019; Wu et al., 2019). We found that 20 ng/mL of exogenous TNF-α can stimulate RA-FLSs and produce a similar effect. It is worth mentioning that 10 or 20 μM of Tan IIA inhibited the increased mRNA expression of IL-6, IL-1β, and IL-8 induced by 20-ng/mL TNF-α. Moreover, only 1 μM of sodium tanshinone IIA sulfonate, a Tan IIA derivate, can decrease IL-6 and IL-1β mRNA expression (Wang Z. et al., 2019). Taken together, these data suggest that Tan IIA acts as an anti-inflammatory in RA by inhibiting the production of pro-inflammatory cytokines, despite the different worked concentrations of Tan IIA or its derivative. Remarkably, Tan IIA did not inhibit TNF-α-induced IL-17 mRNA expression, a result that was similar to those of our previous research on 3′3-Diindolylmethane (DIM) (Du et al., 2019). This may be related to the individual differences of the patients or may indicate that IL-17 production is not related to TNF-α stimulation, and, therefore, it is regulated by other mechanisms. From the ELISA results, we observed that, although there was no increase in TNF-α-induced IL-8, 20μM of Tan IIA suppressed the release of IL-8 by RA-FLSs. Moreover, we also found that Tan IIA inhibited the tendency of IL-1β increase induced by TNF-α, although the basal expression of IL-1β in the blank control group was difficult to detect because it was low.

Previous studies suggested that the expression of MMPs in fibroblasts of synovial joints is responsible for the degradation of synovial collagen in several inflammatory diseases, including RA (Agere et al., 2017). More than fifteen synovial MMPs are expressed in the synovial joints from RA patients, and they fall into three main categories: collagenase, gelatinase, and matrix metalloproteinase (Konttinen et al., 1999). We found that 20 μM of Tan IIA prevented TNF-α-induced mRNA expression of MMP-8 collagenase, MMP-2 and MMP-9 gelatinases, and MMP-3 matrix metalloproteinase. However, we were unable to detect these MMPs at the protein level in the culture supernatant, similar to previous studies (Du et al., 2019). Despite the absence of bands in Western blot and gelatinase analyzes, our data suggest that these MMPs did indeed play an important role in the invasion and migration of RA-FLSs. In addition, we demonstrated that Tan IIA decreased the expression of MMPs in RA-FLSs.

Tan IIA has been reported to affect the proliferation, invasion, and migration of tumor cells through different signaling pathways (Zhang et al., 2018; Liao et al., 2019; Xue et al., 2019). However, such reports left the specific molecular mechanism of Tan IIA in RA-FLSs unknown. We performed network pharmacology analyzes and found some potential pathways for Tan IIA action in the treatment of RA. The integration of the network pharmacology analyses with the experimental *in vitro-*obtained data reveals that Tan IIA can affect three different pathways: MAPK, AKT/mTOR, HIF-1, and NF-kB.

The mitogen-activated protein kinases (MAPK) family is widely conserved among eukaryotes and is responsible for the phosphorylation and dephosphorylation of several key proteins involved in regulatory mechanisms of different cells (Tong et al., 2014). Extracellular signal-regulated kinase (ERK), c-Jun N-terminal kinase (JNK), and P38MAP kinase (p38) are the main members of the MAPK family. These proteins are the main intracellular responders embedded in a highly active signaling flow that is involved in the activation of RA-FLSs (Müller-Ladner et al., 2007; Tong et al., 2014; Bustamante et al., 2017). Several compounds, including sodium tanshinone IIA sulfonate, DIM, and triptolide, have been shown to inhibit MAPK signaling pathway activation by preventing the phosphorylation of p38, JNK, and ERK. Thus, these compounds are able to inhibit the proliferation, metastasis, and invasion of RA-FLSs (Yang et al., 2016; Du et al., 2019; Wang Z. et al., 2019). Our data showed that Tan IIA played an inhibitory role in TNF-α-stimulated p38 and JNK phosphorylation in RA-FLSs but had no significant effect on ERK. Therefore, we suggest that the effect of Tan IIA on proliferation, migration, and invasion in RA-FLSs is mainly mediated by inactivation of p38 and JNK proteins. There is near-consensus that the expression and activation of p38 and JNK in the synovial tissue of RA patients modulate the growth, apoptosis, and differentiation of RA-FLSs. Thus, inflammation and cartilage damage is triggered in the joint cavity of RA patients (Yang et al., 2016; Bustamante et al., 2017).

The PI3K/AKT signaling pathway is involved in the pathogenesis of inflammation (Malemud, 2015), and, therefore, understanding its regulation would be of great benefit for the control of RA (Laragione and Gulko, 2010; Jia et al., 2015). mTOR complex 1 (mTORC1) lies downstream of the PI3K/Akt pathway. The activation of the downstream signaling through AKT-mediated mTORC1 phosphorylation promotes anabolic processes and limits catabolic processes involved in cell growth, proliferation, and metabolism (Liu et al., 2006; Laplante and Sabatini, 2009). Moreover, previous reports have shown that activation of the PI3K/AKT/mTOR pathway appears to be the critical driver of proliferation and anti-apoptosis responses and is a typical feature of inflamed synovial tissue in RA (Garcia et al., 2010). Cytokines, especially TNF-α, in RA-FLS lead to the activation of the PI3K/AKT/mTOR pathway, thereby promoting cell migration and invasion (Karonitsch et al., 2018). Moreover, S6K1 and 4E-BP1 are the two best-characterized mTORC1 substrates, whereby mTORC1 plays the role of an mRNA to protein translator (Wendel et al., 2004). In our data, we found direct evidence that Tan IIA can influence the AKT/mTOR pathway. We showed that Tan IIA blocks activation by TNF-α-stimulated phosphorylation of AKT/mTOR and downstream p70S6K and 4E-BP1. Therefore, these data indicate that Tan IIA has antiproliferative activity and highlight that Tan IIA can be used independently or in combination with other drugs to improve clinical symptoms in RA patients. On the other hand, numerous studies have revealed that autophagy and autophagy-related proteins also participate in the pathogenesis and progress of RA. Furthermore, the mTOR pathway is also involved in autophagy in RA (Li et al., 2017; Wu and Adamopoulos, 2017). Further studies are needed to assess whether Tan IIA can regulate RA-FLS autophagy *via* the AKT/mTOR pathway.

Insufficient oxygen supply appears in the damaged articular cavity in RA pathology and is accompanied by metabolic disorders and pannus hyperplasia, resulting in a hypoxic microenvironment (Fearon et al., 2016; Quiñonez-Flores et al., 2016; Veale et al., 2017). The transcription factors NF-κB and HIFs, in addition to the relevant enzymes, oxygen-sensitive and prolyl hydroxylases, are responsible for responding to the hypoxia signal in the hypoxic microenvironment. In particular, NF-κB and HIFs play key roles in several disorders, including induction of inflammation and angiogenesis and rheumatoid arthritis (Szade et al., 2015; D'Ignazio and Rocha, 2016). Our analysis showed that the HIF-1 pathway may be a potential target for Tan IIA in RA. Based on previous data, we chose NF-κB p65 and HIF-1α as targets to assess their changes in response to hypoxia to highlight the effect of Tan IIA on hypoxia pathways. Our data showed that Tan IIA can actually inhibit HIF-1α expression and TNF-α-stimulated NFκB p65 phosphorylation. Moreover, Tan IIA can also decrease LPS-induced p65 protein expression in PBMCs of RA patients (Tang et al., 2019). Therefore, it can be concluded that Tan IIA may affect RA by suppressing HIF-1α and NF-κB p65 to alleviate damage from hypoxia and the release of proinflammatory cytokines. Nevertheless, the regulatory mechanism of HIF-1α and NF-κB p65 needs further study to be fully revealed.

In conclusion, our data reveal a specific role of Tan IIA on TNF-dependent arthritogenesis. We identified that Tan IIA can inhibit the proliferation, migration, and invasion of RA-FLSs and suppress the release of proinflammatory cytokines and MMPs. We also showed that Tan IIA achieves these effects by affecting the MAPK, AKT/mTOR, HIF-1, and NF-κB signaling pathways. Finally, we present *in vivo* evidence that Tan IIA is able to improve arthritis severity in AIA mice. Therefore, this study highlights the therapeutic role of Tan IIA in the treatment of RA and shows its potential to improve the quality of life of RA patients.

## Data Availability Statement

The raw data supporting the conclusions of this manuscript will be made available by the authors, without undue reservation, to any qualified researcher.

## Ethics Statement

Our study was authorized by the Medical Ethics Committee of Zhujiang Hospital, Southern Medical University. All patients voluntarily signed an informed consent form. All of the animal experiments were conducted with the approval of the Southern Medical University Ethics Committee for Animal Laboratory Research. Animal care and handling procedures abided by the guidelines of ethical regulations for institutional animal care used in the Southern Medical University.

## Author Contributions

Design of the entire study: HD, YiW, and LJ. Experimental studies: HD, YZ, XH, DL, LY, and JW. Network pharmacology analysis: YuW. Animal model construction: YL and XC. Experimental data analysis and statistics: HD, HL, and LJ. Writing and revising the manuscript: HD, QY, YiW, and LJ. All authors read and approved the final manuscript.

## Funding

This work was supported by grants from the National Natural Science Foundation of China (81601397, 81771727, 81102688, and 81401920), the Natural Science Foundation of Guangdong Province (2016A030313624), the Program of Guangdong Innovation and Entrepreneurship training for college students (201812121108), and the Scientific Enlightenment Project of Southern Medical University.

## Conflict of Interest

The authors declare that the research was conducted in the absence of any commercial or financial relationships that could be construed as a potential conflict of interest.
