# Supplementary material for: Tanshinone IIA Suppresses Proliferation and Inflammatory Cytokine Production of Synovial Fibroblasts from Rheumatoid Arthritis Patients Induced by TNF-α and Attenuates the Inflammatory Response in AIA Mice
Source: Front Pharmacol. 2020 May 15;11:568. doi: 10.3389/fphar.2020.00568 (PMC7243269; doi:10.3389/fphar.2020.00568)
Supplement: Supplementary file 1 [file Table_1.docx]

**SUPPLEMENTARY MATERIAL**

**Table S1. The information of RA patients**

**whose tissue samples were used in our research**

| No. of Patients | | Gender | | Age | | RF  (IU/mL) | | ESR | | C-reactin protein  (mg/L) | | ACPA |
| --- | --- | --- | --- | --- | --- | --- | --- | --- | --- | --- | --- | --- |
| 1 | female | | 60 | | 4760 | | 60 | | 127 | | Positive | |
| 2 | female | | 61 | | 795 | | 23 | | 109 | | Positive | |
| 3 | male | | 55 | | 948 | | 103 | | 85.3 | | Positive | |
| 4 | female | | 24 | | 678 | | 90 | | 45.9 | | Positive | |
| 5 | female | | 60 | | 202 | | 56 | | <3.14 | | Positive | |
| 6 | male | | 57 | | 998 | | 29 | | 28.6 | | Positive | |

None of the patients from whom synovial tissues were removed in our research were treated with biologics or JAK inhibitors.

Remark: Rheumatoid factor (RF); Erythrocyte sedimentation rate (ESR); Anti-cyclic citrullinated peptide antibodies (ACPA)
