# Supplementary material for: Tanshinone IIA Suppresses Proliferation and Inflammatory Cytokine Production of Synovial Fibroblasts from Rheumatoid Arthritis Patients Induced by TNF-α and Attenuates the Inflammatory Response in AIA Mice
Source: Front Pharmacol. 2020 May 15;11:568. doi: 10.3389/fphar.2020.00568 (PMC7243269; doi:10.3389/fphar.2020.00568)
Supplement: Supplementary file 2 [file Table_2.docx]

**SUPPLEMENTARY MATERIAL**

**Tabel S2. The primers of human cytokines and MMPs**

**for mRNA expression assay**

| Gene Name |  | Sequence | Number of bases(bps) |
| --- | --- | --- | --- |
| IL-6 | Forward | AGTGAGGAACAAGCCAGAGC | 20 |
|  | Reverse | AGCTGCGCAGAATGAGATGA | 20 |
| il-8 | Forward | AGAAGTTTTTGAAGAGGGCTGAGA | 25 |
|  | Reverse | AGTTTCACTGGCATCTTCACTGATT | 25 |
| il-1β | Forward | CCACCTCCAGGGACAGGATA | 20 |
|  | Reverse | AACACGCAGGACAGGTACAG | 20 |
| IL-17 | Forward | CTGTCCCCATCCAGCAAGAG | 20 |
|  | Reverse | AGGCCACATGGTGGACAATC | 20 |
| mmp-2 | Forward | TCGCCCATCATCAAGTTCCC | 20 |
|  | Reverse | GGGCAGCCATAGAAGGTGTT | 20 |
| MMP-3 | Forward | TCCGACACTCTGGAGGTGAT | 20 |
|  | Reverse | ACTTCGGGATGCCAGGAAAG | 20 |
| mmp-8 | Forward | ATGTGACGGGGAAGCCAAAT | 20 |
|  | Reverse | AAAACCACCACTGTCAGGCA | 20 |
| mmp-9 | Forward | GGACAAGCTCTTCGGCTTCT | 20 |
|  | Reverse | TCGCTGGTACAGGTCGAGTA | 20 |
